# Dioscin promotes osteoblastic proliferation and differentiation via Lrp5 and ER pathway in mouse and human osteoblast-like cell lines

**DOI:** 10.1186/1423-0127-21-30

**Published:** 2014-04-17

**Authors:** Chunfang Zhang, Jinyong Peng, Shan Wu, Yue Jin, Fan Xia, Changyuan Wang, Kexin Liu, Huijun Sun, Mozhen Liu

**Affiliations:** 1 Department of Clinical Pharmacology, College of Pharmacy, Dalian Medical University, Dalian, China; 2 Department of Orthopaedics, First Affiliated Hospital, Dalian Medical University, Dalian, China

**Keywords:** Dioscin, Proliferation, Differentiation, Lrp5, ER, Osteoporosis

## Abstract

**Background:**

Dioscin, a typical steroid saponin, is isolated from Dioscorea nipponica Makino and Dioscorea zingiberensis Wright. It has estrogenic activity and many studies have also reported that dioscorea plants have an effect in preventing and treating osteoporosis. However, the molecular mechanisms underlying their effect on osteoporosis treatment are poorly understood. Therefore, the present study aims to investigate the mechanism (s) by which dioscin promotes osteoblastic proliferation and differentiation in mouse pre-osteoblast like MC3T3-E1 cells and human osteoblast-like MG-63 cells.

**Results:**

We found that dioscin (0.25 μg/ml, 0.5 μg/ml, and 1.0 μg/ml) promoted MC3T3-E1 cells and MG-63 cells proliferation and differentiation dose dependently. Western blot analysis results showed that estrogen receptor α (ER-α), estrogen receptor β (ER-β), β-catenin and Bcl-2 protein expression increased after MC3T3-E1 cells were treated with dioscin. Quantitative reverse transcription-polymerase chain reaction (RT-PCR) analysis indicated that dioscin could increase the ratio of osteoprotegerin (OPG)/receptor activator of NF-κB ligand (RANKL) and up-regulate the level of Lrp5 and β-catenin. And by RNA interference analysis, we proved that the effect of dioscin increasing the ratio of OPG/RANKL was dependent on Lrp5 pathway. In addition, we also found that these effects of dioscin were abolished by ICI 182, 780 (100 nM), an antagonist of ER, indicating that an ER signaling pathway was also involved. We also found that dioscin (0.25 μg/ml, 0.5 μg/ml, and 1.0 μg/ml) induced MG-63 cells proliferation and differentiation in a dose-dependent manner. Western blot analysis results indicated that ER-α, ER-β and β-catenin protein expression increased after MG-63 cells were treated with dioscin.

**Conclusions:**

The current study is the first to reveal that dioscin can promote osteoblasts proliferation and differentiation via Lrp5 and ER pathway.

## Background

Osteoporosis is a universal major public health problem which is defined conceptually as a skeletal disorder characterized by low bone mass, deterioration of bone tissues and increased risk of fracture [1,2]. Bone metabolic balance is maintained by the balance of bone resorption and bone formation, which depends on the interactions between osteoblasts and osteoclasts. And bone metabolic diseases are caused by an imbalance between the bone formation and bone resorption [1,3]. Osteoblasts, bone-forming cells, are controlled by hormonal and local factors such as the canonical Wnt/Lrp5/β-catenin signaling pathway [4]. And the canonical Wnt/Lrp5/β-catenin signaling pathway plays an essential role in bone mass accrual, maintenance, and regulation [5]. Wnt glycoproteins bind to the receptor frizzled (Fzd) and their co-receptor low-density lipoprotein receptor-related protein 5/6 (Lrp5/6) complex, leading to stabilization and accumulation of β-catenin in the cytoplasm [5,6]. Substantial genetic data demonstrate Lrp5 as a regulator of bone density. And numerous studies reported that Lrp5 associates with multiple abnormal bone phenotypes, including osteoporosis-pseudoglioma (OPPG), high bone mass (HBM) and autosomal recessive osteopetrosis [7]. β-catenin is an essential mediator of signals emanating from Lrp5 in osteoblasts and can promote osteoblasts survival and differentiation through both Wnt-dependent and independent events [7]. Thus, the pathways play a crucial role in bone remodeling.

Osteoporosis can occur at any age and in any racial or ethnic group, though more common in post-menopausal women. It is known that estrogen plays a significant role in the regulation of bone remodeling and maintenance of formation [8,9] and many studies have investigated that loss of estrogen induces reduction of bone mass and results in post-menopausal osteoporosis [8,10]. Estrogens perform their physiological effects on target tissues through combining with estrogen receptors, and two subtypes of estrogen receptor (ER), ER-α and ER-β, have been identified in osteoblasts and osteoclasts. Estrogen acts on skeleton by the two classical estrogen receptors, both ER-α and ER-β. And several studies also demonstrate that estrogens may prevent osteoporosis by regulating bone formation [10,11]. Thus, to date, the main treatment for postmenopausal osteoporosis is hormone replacement therapy (HRT) [9,12]. However, compliance with HRT is poor because of the increased risks of breast and uterine cancers associated with long term of HRT [9,12]. So newer drugs which can overcome the concerns of HRT are of great interest to both clinicians and patients. Statins, which are widely used for hyperlipidemia treatment, can promote bone formation and suppress bone resorption [13]. And previous study has reported that statins can also promote estrogen receptors expression, but the side effects limit the use of it in treating osteoporosis [12]. Dioscin is an active ingredient identified in edible medicinal plants such as Dioscorea nipponica Makino and Dioscorea zingiberensis Wright [14]. Previous pharmacological studies have demonstrated that dioscin not only has anti-tumor [15] and anti-fungal activities [16], but also can regulate hyperlipidemia [17] and protect liver [14]. And related studies have reported that dioscorea plants have a role for treatment of osteoporosis and perform estrogen-like effects [18,19]. Qu et al. had reported that dioscin inhibits osteoclast differentiation and bone resorption though down-regulating the Akt signaling pathway [20]. Statins are specific inhibitors of 3-hydroxy-3-methylglutaryl coenzyme A (HMG-CoA) reductase, a rate limiting enzyme involved in the cholesterol synthesis pathway and statins have also been reported to possess anabolic effects on bone [21]. In the present studies, we investigated the mechanism by which dioscin prevents osteoporosis using lovastatin as a positive control. We found that dioscin promoted proliferation and differentiation of osteoblasts. And this might be related to the effects of dioscin up-regulating ERs (ER-α and ER-β) and β-catenin protein expression and stimulating Lrp5, β-catenin mRNA expression levels and increasing the ratio of OPG/ RANKL. Our results, for the first time revealed the multiple working mechanism of dioscin on the prevention and therapy of osteoporosis.

## Methods

MC3T3-E1 cells and human osteoblast-like MG-63 cells were purchased from Insitute of Biochemistry and Cell Biology, CAS, Shanghai, China. Dulbecco’s modified Eagle’s medium (DMEM) was purchased from GIBCO, USA. Fetal bovine serum (FBS) were obtained from Tianjin Haoyang Biologicals Technology Co., Ltd.. Dioscin with purity of over 98% was isolated from Dioscorea nipponica Makino using the method reported in previous study [22] and it was dissolved in dimethyl sulfoxide (DMSO). Lovastatin, which was used as positive control, was purchased from Dalian Lvzhu Biologicals Technology Co., Ltd.. ALP activity assay kit was purchased from Nanjing Jiancheng Bioengineering Institute, Nanjing, China. Western & IP Cell lysate, GAPDH antibody, BCA Protein Assay Kit and BeyoECL Plus were purchased from Beyotine Institute of Biotechnology. Estrogen Receptor-α (ERα), Estrogen Receptor-β (ERβ) PolyClonal Antibody and Bcl-2 PolyClonal Antibody were purchased from Proteintech Group, Inc (Wuhan). PrimeScript® RT regent Kit With gDNA Eraser (Perfect Real Time), SYBR® Premix Ex TaqTM (Tli RNaseH Plus) and RNAiso Plus were purchased from TaKaRa Biotechnology (Dalian) Co., Ltd. RNAi Oligo and Lipofectamine 2000™ were purchased from Invitrogen. β-catenin MonoClonal, PolyClonal Antibody and ICI 182, 780 was purchased from Santa cruz.

### Cells culture

MC3T3-E1 cells and MG-63 cells were maintained in DMEM supplemented with 10% FBS, 100 U/ml penicillin and 100 mg/ml streptomycin. Cells were cultured at 37°C in a humidified atmosphere of 5% CO_2_. This medium was changed every two to three days.

### Cell proliferation assay

Cell proliferation was evaluated with the MTT (3-dimethylthiazol-2-y- [4,5]-2, 5-diphenyltetrazolium bromide) method. MC3T3-E1 cells and MG-63 cells were seeded in 96-well culture plates and cultured overnight in an incubator. The medium was removed and cells were treated with dioscin (0.25 μg/ml, 0.5 μg/ml and 1.0 μg/ml) for 24 h, 48 h and 72 h. Then, MTT (10 μl per well, 5 mg/ml) solution was added in each well and incubated at 37°C for 4 h. The absorbance was measured at 570 nm by the Enzyme standard instrument.

### ALP activity assay

MC3T3-E1 cells and MG-63 cells were seeded in 24-well culture plates. MC3T3-E1 cells and MG-63 cells were treated with dioscin (0.25 μg/ml, 0.5 μg/ml and 1.0 μg/ml) or lovastatin (0.04 μM) for 72 h. The cell monolayer was gently washed twice with iced PBS. Cells were lyzed with 0.2% TritonX-100 and the lysate was centrifuged at 14, 000 × g for 10 min at 4°C. The clear supernatant was used for the measurement of ALP activity and total protein concentration using an ALP activity assay kit and a BCA-protein assay kit.

### Mineralization assay

The mineralization nodules were measured by von Kossa staining. MC3T3-E1 cells were seeded in 6-well culture plates. Then cells were treated with dioscin (0.25 μg/ml, 0.5 μg/ml and 1.0 μg/ml) or lovastatin (0.04 μM) for 72 h. The medium was removed and cells were cultured with the medium supplemented with Vitamin C and β-glycerol phosphate disodium salt pentahydrate at final concentrations of 50 μg/ml and 10 mM at 37°C for 17 days. The cell monolayer was stained following the reference [23]. The cells were fixed with 4% paraformaldehyde and incubated using 5% sodium thiosulfate for 30 min. Then, 2 ml of freshly prepared 1% silver nitrate was added to wells, which were incubated under UV light for 30 min. The wells were rinsed with distilled water and fixed using 5% sodium thiosulfate for 2 min, then rinsed thoroughly with distilled water to terminate the reaction. Then, wells were redyed using 1% neutral red for 10 min and rinsed thoroughly with distilled water. The formed nodules were photographed with a Canon camera. We randomly chose five views and recorded mineralization nodules.

### Western blot analysis

The expression of ER-α, ER-β and Bcl-2 proteins was detected by Western blot. MC3T3-E1 cells and MG-63 cells were treated with dioscin (0.25 μg/ml, 0.5 μg/ml and 1.0 μg/ml) or lovastatin (0.04 μM) for 72 h or 24 h and then the cell monolayer was gently washed twice with iced PBS. The cells were prepared with 100 μl Western & IP Cell lysate on ice for 30s, then the lysate was centrifuged at 12, 000 × g for 10 min at 4°C. The centrifuged supernatant was collected, and the total protein concentration was measured using a BCA-protein assay kit with BSA as the standard. Proteins were mixed with 6 × sodium dodecyl sulphate (SDS) sample buffer. Equal amounts of protein (20 μg) was resolved on a 15% SDS-polyacrylamide gel, followed by blotting to a polyvinylidene fluoride (PVDF) membrane. The membrane was blocked by 5% milk in TTBS for 2 h at 37°C. Then the membrane was incubated overnight at 4°C with ER-α polyclonal antibody (1:600), ER-β polyclonal antibody (1:1000), β-catenin monoclonal antibody (1:1000), β-catenin polyclonal antibody (1:1000) and Bcl-2 polyclonal antibody (1: 1000). The following day, the membrane was incubated with Peroxidase-Conjugated AffiniPure goat anti-rabbit IgG (H + L) for 2 h at room temperature. Finally, the membrane was visualized by ECL Plus as specified by the manufacturer.

### RNA extraction and quantitative reverse transcription- polymerase chain reaction (RT-PCR)

The expressions of Lrp5, β-catenin, OPG, RANKL mRNA were detected by RT-PCR. Then MC3T3-E1 cells were treated with various concentrations of dioscin (0.25 μg/ml, 0.5 μg/ml and 1.0 μg/ml) or lovastatin (0.04 μM). Total RNA was isolated using RNAiso Plus according to the manufacturer’s instructions. The concentration and purity of the RNA were determined by measuring the absorbance at 260 nm and 280 nm. Total RNA was reverse-transcribed in 10 μL of a reaction mixture that contained gDNA Eraser Buffer, gDNA Eraser, RNase Free dH_2_O and 1.0 μL total RNA according at 42°C for 2 min. PCR was carried out in a 20 μL reaction mixture containing SYBR® Premix Ex Taq™, specific primers (10 μM each, Table 1), ROX Referenxe DyeII, dH_2_O and 2.0 μL of cDNA template. The PCR were performed using the following cycle parameters: one cycle of 95°C for 30 s, and 40 cycles of 95°C for 5 s, 60°C for 30 s. The target gene transcripts in each sample were normalized on the basis of its GAPDH. Primers for GAPDH [24], Lrp5, β-catenin [25], OPG and RANKL are listed in the Table 1.

**Table 1:** Nucleotide sequences of primers used for real time PCR detection

***Gene***	***Primer sequence (5’ to 3’)***
Lrp5	Forward: ctgccaggatcgctctgatg
	Reverse: acactgttgcttgatgaggacacac
β-catenin	Forward: gccacaggattacaagaagc
	Reverse: ccaccagagtgaaaagaacg
OPG	Forward: ttacctggagatcgaattctgcttg
	Reverse: gtgctttcgatgaagtctcagctg
RANKL	Forward: gcagcatcgctctgttcctgta
	Reverse: cctgcaggagtcaggtagtgtgtc
GAPDH	Forward: gaccacagtccatgccatcac
	Reverse: gctgttgaagtcgcaggagac

### RNA interference of Lrp5 gene

The RNA duplexes targeting the sequence of mouse Lrp5 (NM_008513) and scrambled control oligonucleotide were synthesized by Invitrogen. Cultured MC3T3-E1 cells were transfected with the siRNA and the control siRNA according to manufacturer’s instructions. Four microliters of Lipofectamine 2000™ and 40 nM small interfering RNA or 40 nM control oligonucleotide were used for transfection. The result of knockdown was validated by RT-PCR analysis. The sequences of siRNA-Lrp5 and control siRNA are listed in the Table 2.

**Table 2:** **The sequences of siRNA**-**Lrp5 and control siRNA**

***Gene***	***siRNA Sequence (5’ to 3’)***
Lrp5	Forward: cccggaagaucauuguagatt
Control	Reverse: cuuggcucuuucucugucctt
	Forward: uucuccgaacgugucacgutt
	Reverse: acgugacacguucggagaatt

### Statistics

All assays were repeated in three independent experiments. The results were expressed as the mean ± SD. Statistical analysis to compare results between groups was conducted by one-way analysis of variance (ANOVA). All statistical tests were 2-tailed, and P < 0.05 or P < 0.01 was considered significant.

## Results

### Effects of dioscin on MC3T3-E1 cell and MG-63 cell proliferation

**Figure 1:**
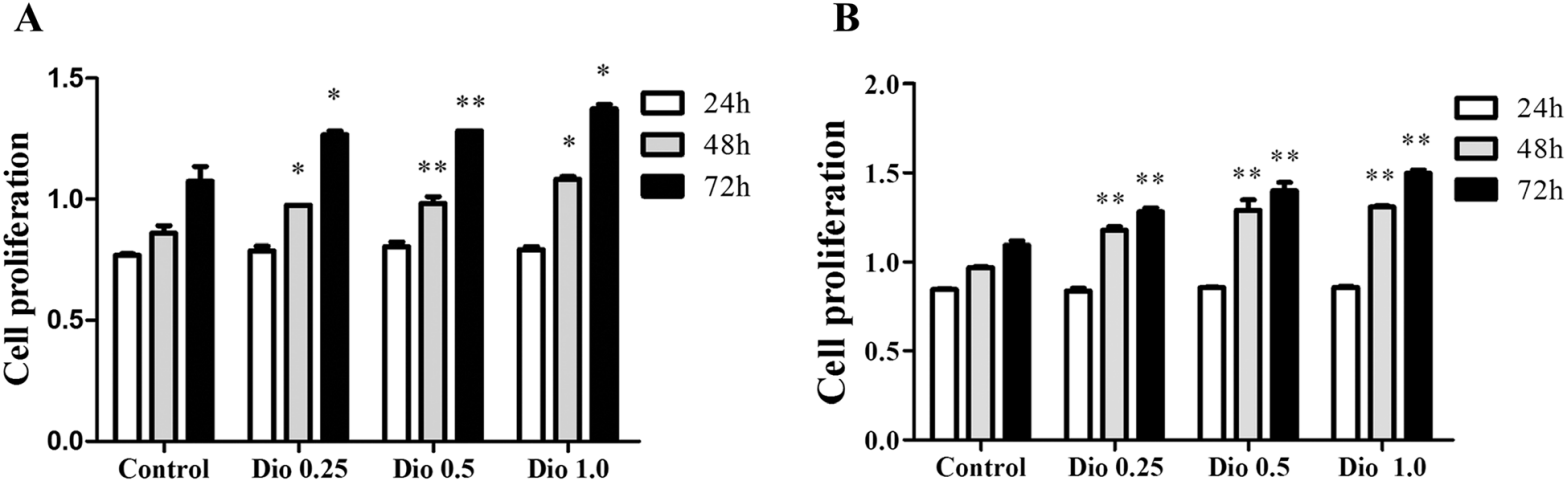
**Effects of dioscin on cell proliferation in MC3T3**-**E1 cells and MG-63 cells.** MC3T3-E1 cells **(A)** and MG-63 cells **(B)** were treated with vehicle (Control), dioscin (0.25 μg/ml, 0.5 μg/ml and 1.0 μg/ml) or lovastatin (0.04 μM) for 24 h, 48 h and 72 h. Cell proliferation was assessed by MTT assay. Results were obtained from three independent experiments and were expressed as mean ± SD. *P < 0.05; **P < 0.01 *vs* Control (Dio 24 h *vs* Control 24 h; Dio 48 h *vs* Control 48 h; Dio 72 h *vs* Control 72 h).

The process of bone formation includes proliferation of osteoprogenitor cells, maturation of extracellular matrix and deposition of minerals in the matrix [26]. MC3T3-E1 cells (Figure 1A, P < 0.05 or P < 0.01) and MG-63 cells (Figure 1B, P < 0.01) were incubated with dioscin of various concentrations and cell growth was measured with MTT assays to evaluate the rate of cell proliferation. The results showed that dioscin, concentration of 0.25 μg/ml, 0.5 μg/ml and 1.0 μg/ml, promoted MC3T3-E1 cells and MG-63 cells proliferation in 48 h and 72 h significantly in a concentration-dependent manner compared with control cells (Figure 1, P < 0.05 or P < 0.01).

### Effect of dioscin on expression of Bcl-2 protein in MC3T3-E1 cells

**Figure 2:**
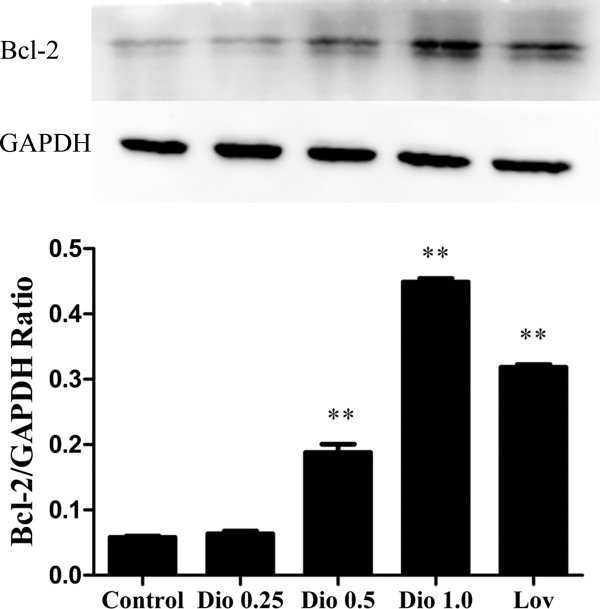
**Effect of dioscin on the expression level of Bcl-2 protein in MC3T3-E1 cells.** Cells were treated with vehicle, different concentrations of dioscin (0.25 μg/ml, 0.5 μg/ml and 1.0 μg/ml) or lovastatin (0.04 μM) for 24 h, and then the expression level of Bcl-2 protein was examined by Western blot. Results were obtained from three independent experiments and were expressed as mean ± SD. **P < 0.01 *vs* Control.

Bcl-2, an anti-apoptotic protein, plays an important role in the initiation and execution of the intrinsic pathway of apoptosis [27]. Therefore, Bcl-2 protein expression level was analyzed to study the effect of dioscin on the inhibitory effect of osteoblastic apoptosis in MC3T3-E1 cells. We analyzed the expression of Bcl-2 protein following 24 h exposure to various concentrations of dioscin by Western blot. The result showed that dioscin increased Bcl-2 protein expression in a concentration-dependent manner (Figure 2, P < 0.01).

### Effects of dioscin on ALP activity in MC3T3-E1 cells and MG-63 cells

**Figure 3:**
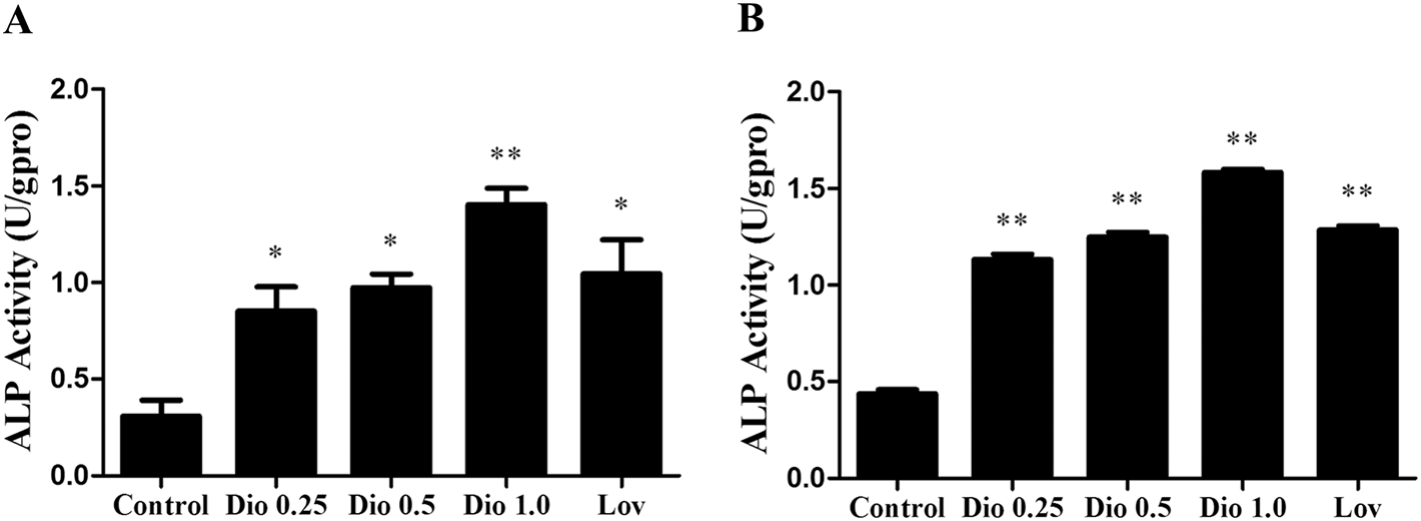
**Effects of dioscin on ALP activity in MC3T3**-**E1 cells and MG**-**63 cells.** MC3T3-E1 cells **(A)** and MG-63 cells **(B)** were treated with vehicle, dioscin (0.25 μg/ml, 0.5 μg/ml and 1.0 μg/ml) or lovastatin (0.04 μM) for 72 h. The lysates were used for analysis of ALP activity. Results were obtained from three independent experiments and were expressed as mean ± SD. *P < 0.05; **P < 0.01 *vs* Control.

Since the appearance of ALP activity is represented as an early biochemical marker for osteoblasts differentiation [26], we examined the ALP activity of MC3T3-E1 cells (Figure 3A, P < 0.05 or P < 0.01) and MG-63 cells (Figure 3B, P < 0.01) in response to dioscin. We found that dioscin treatment could result in an obvious increase in ALP activity compared with respective control cells, and the effect was dose-dependent.

### Effect of dioscin on the mineralization in MC3T3-E1 cells

**Figure 4:**
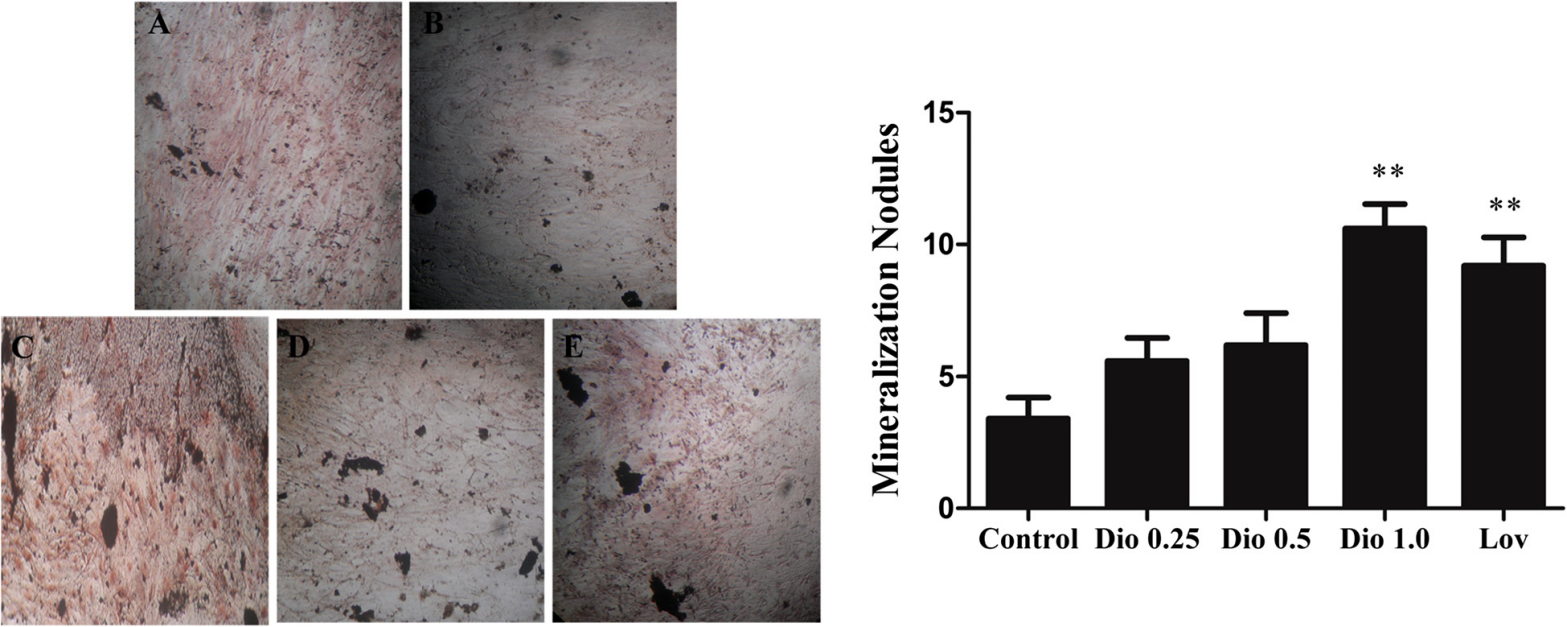
**Effects of dioscin on mineralization in MC3T3**-**E1 cells.** Cells were treated with vehicle, dioscin (0.25 μg/ml, 0.5 μg/ml and 1.0 μg/ml) or lovastatin (0.04 μM) for 72 h. The medium was removed and cells were cultured with the medium supplemented with Vitamin C and β-glycerol phosphate disodium salt pentahydrate at final concentrations of 50 μg/ml and 10 mM at 37°C for 17 days. And the mineralized nodules were analyzed by von Kossa staining. **(A)** Control; **(B)** Dio 0.25; **(C)** Dio 0.5; **(D)** Dio 1.0; **(E)** Lov. **P < 0.01 *vs* Control.

To examine the effect of dioscin on mineralization, we evaluated whether dioscin treatment could promote the formation of mineralization nodule in MC3T3-E1 cells. Extracellular matrix calcium deposits for mineralized nodule formation were stained with von Kossa and the calcified nodules appeared black color. Mineralization by MC3T3-E1 cells occurred within 20 days culture. Dioscin stimulated the formation of mineralization nodule in a concentration-dependent manner and higher concentration (1.0 μg/ml) of dioscin or lovastatin resulted in a significant increase compared with control cells (Figure 4, P < 0.01).

### Effect of dioscin on the ratio of OPG/ RANKL mRNA in MC3T3-E1 cells

**Figure 5:**
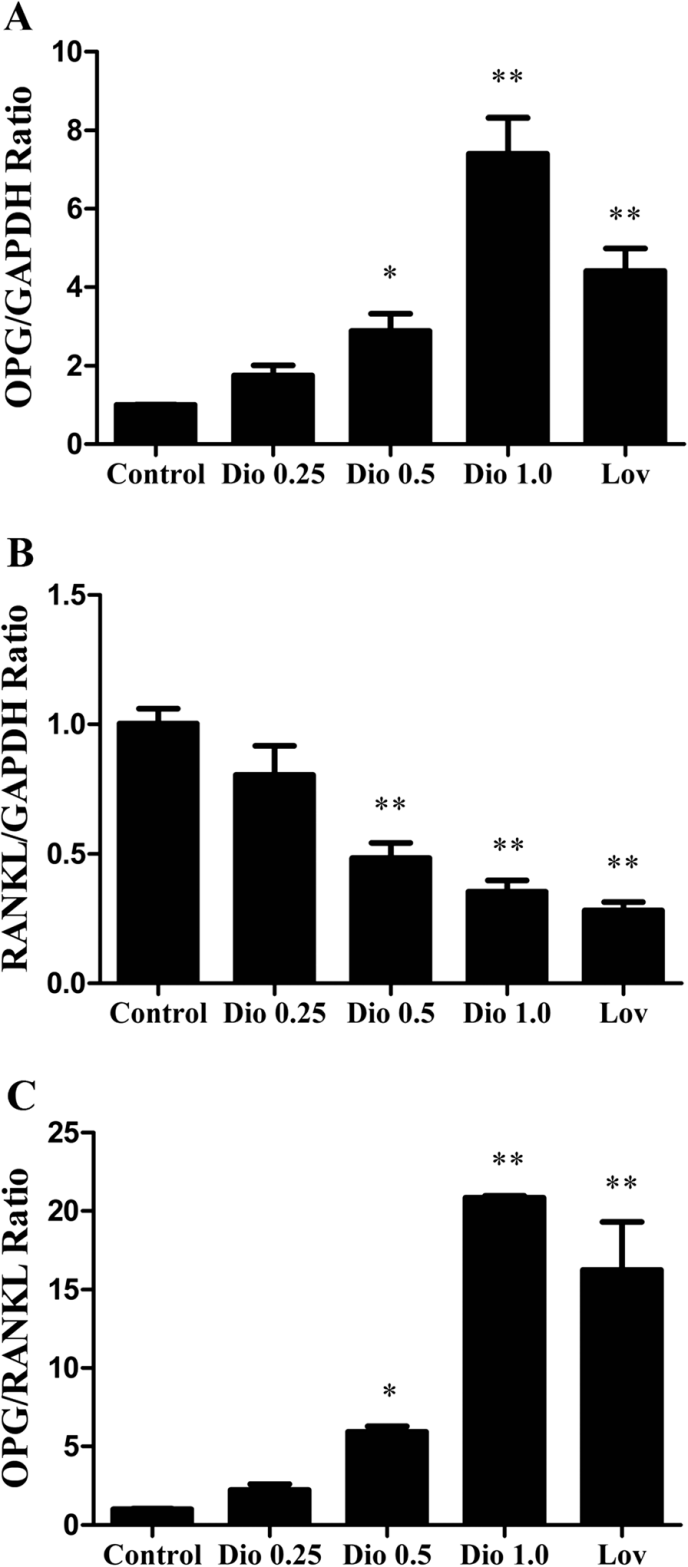
**Effect of dioscin on the ratio of OPG/RANKL mRNA in MC3T3**-**E1 cells.** MC3T3-E1 cells were treated with vehicle, different concentrations of dioscin (0.25 μg/ml, 0.5 μg/ml and 1.0 μg/ml) or lovastatin (0.04 μM) for 72 h, and then the expressions of OPG **(A)** and RANKL **(B)** mRNA were examined by RT-PCR. And the ratio of OPG/ RANKL mRNA **(C)** was obtained. Results were obtained from three independent experiments and were expressed as mean ± SD. *P < 0.05; **P < 0.01 *vs* Control.

The balance between OPG and RANKL is important to the regulation of bone remodeling and the ratio of OPG/RANKL mRNA expression in osteoblastic cells is an essential factor in bone resorption [24,28]. Cells were treated with dioscin (0.25 μg/ml, 0.5 μg/ml and 1.0 μg/ml) or lovastatin (0.04 μM) for 72 h and then total RNA was isolated to evaluate the effect of dioscin on the ratio of OPG/RANKL mRNA in MC3T3-E1 cells. As shown in Figure 5, dioscin not only obviously increased OPG mRNA expression in MC3T3-E1 cells at concentrations (0.5 μg/ml and 1.0 μg/ml) tested (Figure 5A), but also obviously decreased RANKL mRNA expression at tested concentrations (0.5 μg/ml and 1.0 μg/ml) (Figure 5B). The effects of dioscin or lovastatin on the ratio of OPG/ RANKL mRNA expression in MC3T3-E1 cells were shown in Figure 5C. The results clearly showed that diocsin or lovastatin could increase the ratio of OPG/ RANKL mRNA expression dramatically, suggesting that dioscin might regulate the process of osteoblastogenesis by its actions on OPG and RANKL expressions.

### Effects of dioscin on expression of ER-α and ER-β in MC3T3-E1 cells and MG-63 cells

**Figure 6:**
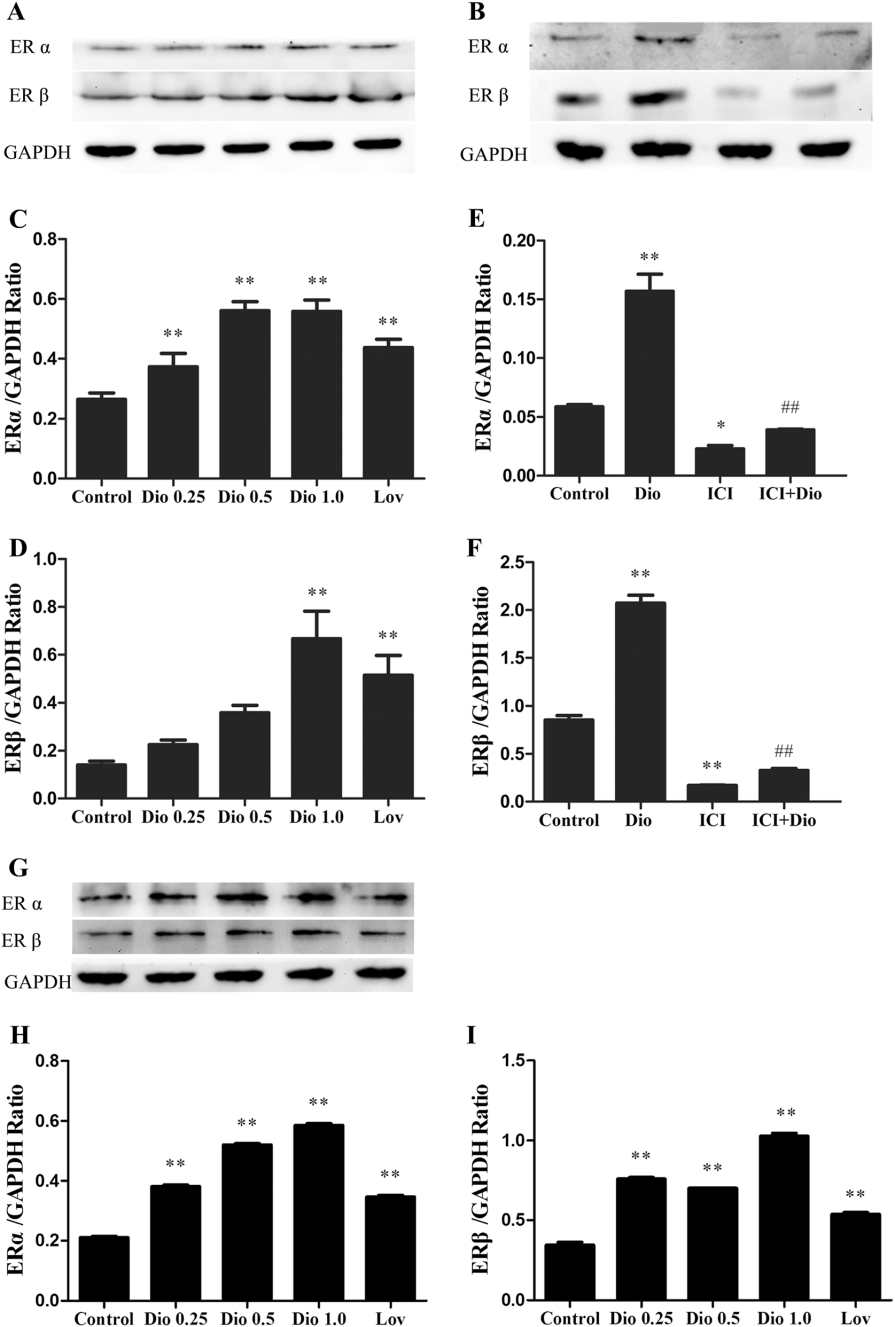
**Effects of dioscin on the expression levels of ER-α and ER**-**β proteins in MC3T3-E1 cells and MG-63 cells.** MC3T3-E1 cells and MG-63 cells were treated with vehicle, different concentrations of dioscin (0.25 μg/ml, 0.5 μg/ml and 1.0 μg/ml) or lovastatin (0.04 μM) for 72 h. Then the expression levels of ER-α **(A, C)** and ER-β **(A, D)** proteins in MC3T3-E1 cells and the proteins expression levels of ER-α **(G, H)** and ER-β **(G, I)** in MG-63 cells were examined by Western blot. Pretreatment of MC3T3-E1 cells with ICI 182, 780 (100 nM) for 1 h, cells were treated with dioscin (1.0 μg/ml) for 72 h, and then the expression levels of ER-α **(B, E)** and ER-β **(B, F)** proteins were examined by Western blot. Results were obtained from three independent experiments and expressed as mean ± SD. *P < 0.05; **P < 0.01 *vs* Control.

Dioscorea nipponica Makino and Dioscorea zingiberensis Wright have estrogenic activity and estrogen plays an important role in the regulation of bone remodeling and maintenance of formation [9,19], therefore we examined the expression levels of ER-α and ER-β in MC3T3-E1 cells and MG-63 cells in response to dioscin by Western blot. The results revealed that compared with control cells the expression level of ER-α in MC3T3-E1 cells was up-regulated significantly in a dose-dependent manner after the cells were treated with dioscin for 72 h (Figure 6A, C, P < 0.01). Dioscin of 1.0 μg/ml showed a significant effect to increase the expression level of ER-β protein compared with control cells (Figure 6A, D, P < 0.01). However, after pretreatment by the specific ER antagonist ICI 182, 780 for 1 h, the expression of ER-α (Figure 6B, E, P < 0.05) and ER-β (Figure 6B, F, P < 0.01) protein was reduced compared with control cells, and the effect of dioscin up-regulating ER-α and ER-β protein level in MC3T3-E1 cells decreased significantly compared with dioscin group cells (Figure 6B, E and Figure 6B, F, P < 0.01). And our results also indicated that dioscin could up-regulated obviously the protein expression levels of ER-α (Figure 6G, H, P < 0.01) and ER-β (Figure 6G, I, P < 0.01) in MG-63 cells. Therefore, our results demonstrate that ER-pathway is involved in dioscin-mediated effects on osteoblasts proliferation and differentiation.

### Effect of dioscin on expression of Lrp5 and β-catenin mRNA levels in MC3T3-E1 cells

**Figure 7:**
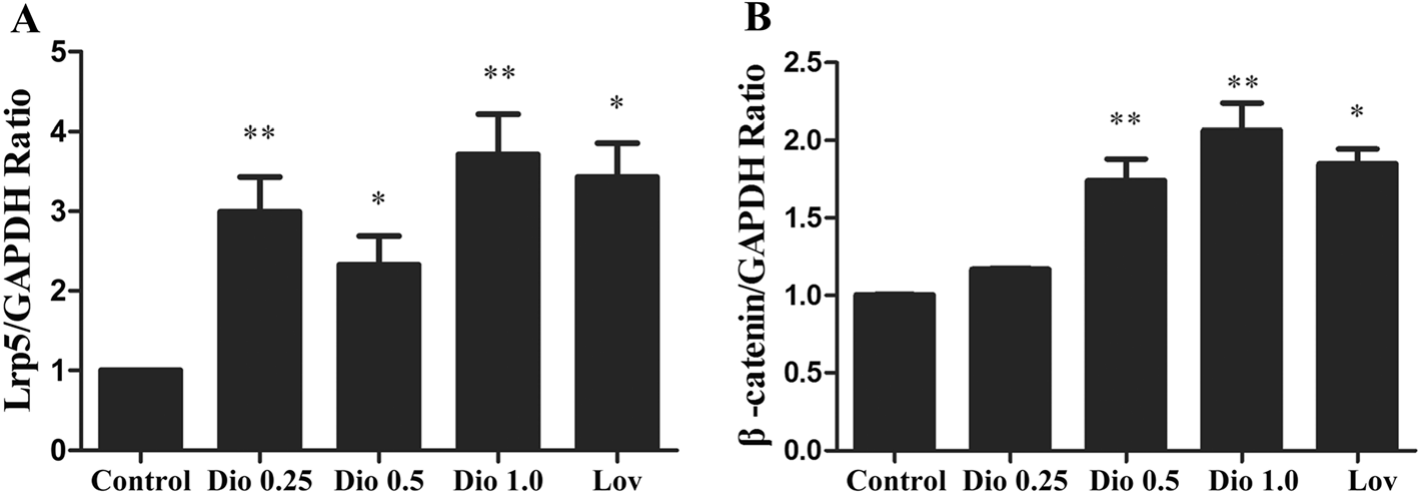
**Effects of dioscin on the expression levels of Lrp5 and β-catenin mRNA in MC3T3-E1 cells.** MC3T3-E1 cells treated with vehicle, dioscin (0.25 μg/ml, 0.5 μg/ml and 1.0 μg/ml) or lovastatin (0.04 μM) for 48 h. The expression levels of Lrp5 **(A)** and β-catenin **(B)** mRNA were examined by RT-PCR. Results were obtained from three independent experiments and were expressed as mean ± SD. *P < 0.05; **P < 0.01 *vs* Control.

Lrp5, a critical co-receptor for Wnt signaling pathway, has been identified as an important contributor to bone health. β-catenin acts downstream of Lrp5 and also plays an important role in bone formation [26,29]. Therefore, whether this pathway is involved in the effects of dioscin on osteoblasts was detected. Cells were treated with various concentrations of dioscin or lovastatin for 48 h. Total RNA was isolated to study the effect of dioscin on Lrp5 and β-catenin mRNA expression levels in MC3T3-E1 cells. As shown in Figure 7, compared with control group, dioscin not only increased Lrp5 mRNA expression significantly at all concentrations (0.25 μg/ml, 0.5 μg/ml and 1.0 μg/ml) (Figure 7A, P < 0.05 or P < 0.01), but also up-regulated β-catenin mRNA expression level obviously at concentrations of 0.5 μg/ml and 1.0 μg/ml (Figure 7B, P < 0.01). And the results also clearly demonstrated that lovastatin (0.04 μM) could induce a significant up-regulation on the expression levels of Lrp5 and β-catenin mRNA in MC3T3-E1 cells (P < 0.05 or P < 0.01).

### Effects of dioscin on expression of β-catenin protein in MC3T3-E1 cells and MG-63 cells

**Figure 8:**
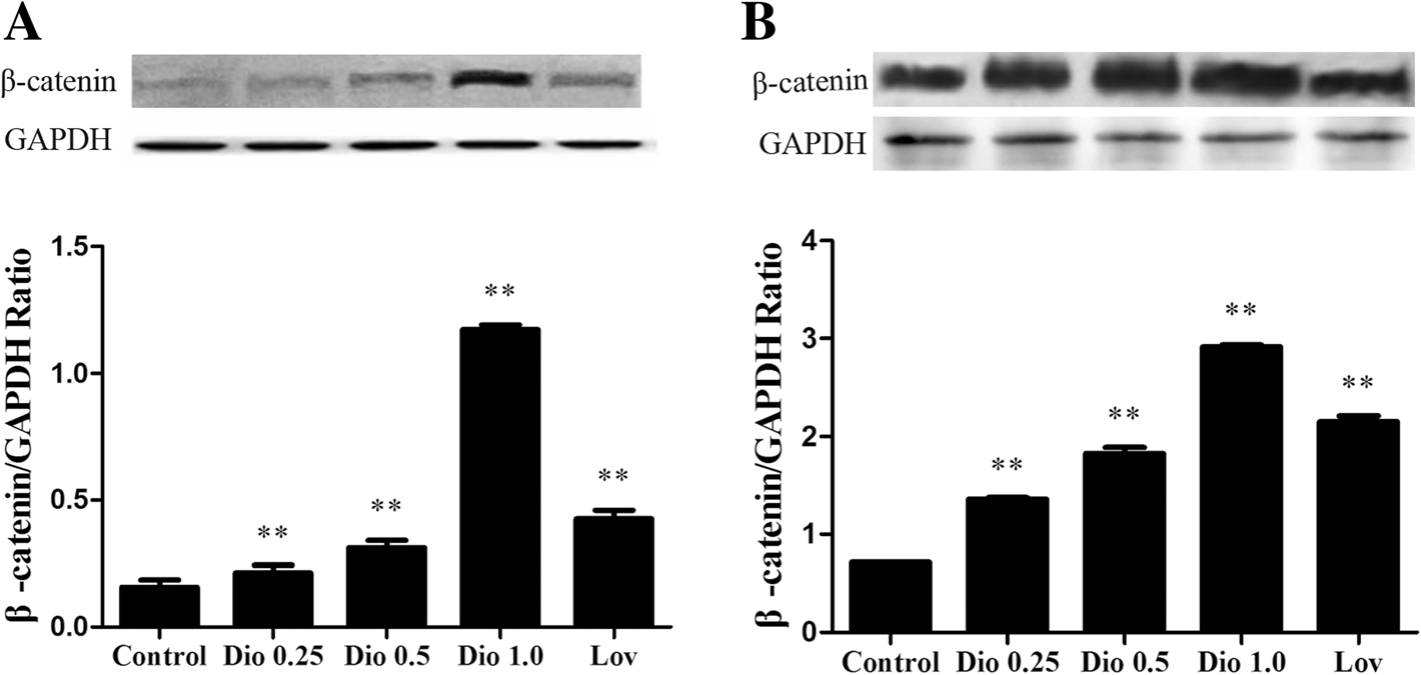
**Effects of dioscin on expression of β-catenin protein in MC3T3**-**E1 cells and MG-63 cells.** MC3T3-E1 cells and MG-63 cells were treated with vehicle, different concentrations of dioscin (0.25 μg/ml, 0.5 μg/ml and 1.0 μg/ml) or lovastatin (0.04 μM) for 72 h. Then the expression levels of β-catenin proteins in MC3T3-E1 cells **(A)** and MG-63 cells **(B)** were examined by Western blot. Results were obtained from three independent experiments and expressed as mean ± SD. **P < 0.01 *vs* Control.

Then we examined the expression levels of β-catenin protein in MC3T3-E1 and MG-63 cells in response to dioscin treatment by Western blot. The results revealed that compared with their respective control cells, the expressions levels of β-catenin in MC3T3-E1 cells (Figure 8A, P < 0.01) and MG-63 cells (Figure 8B, P < 0.01) were up-regulated significantly in a dose-dependent manner after the cells were treated with dioscin for 72 h.

### The stimulating activity of dioscin on the ratio of OPG/ RANKL mRNA was dependent on the Lrp5 pathway

**Figure 9:**
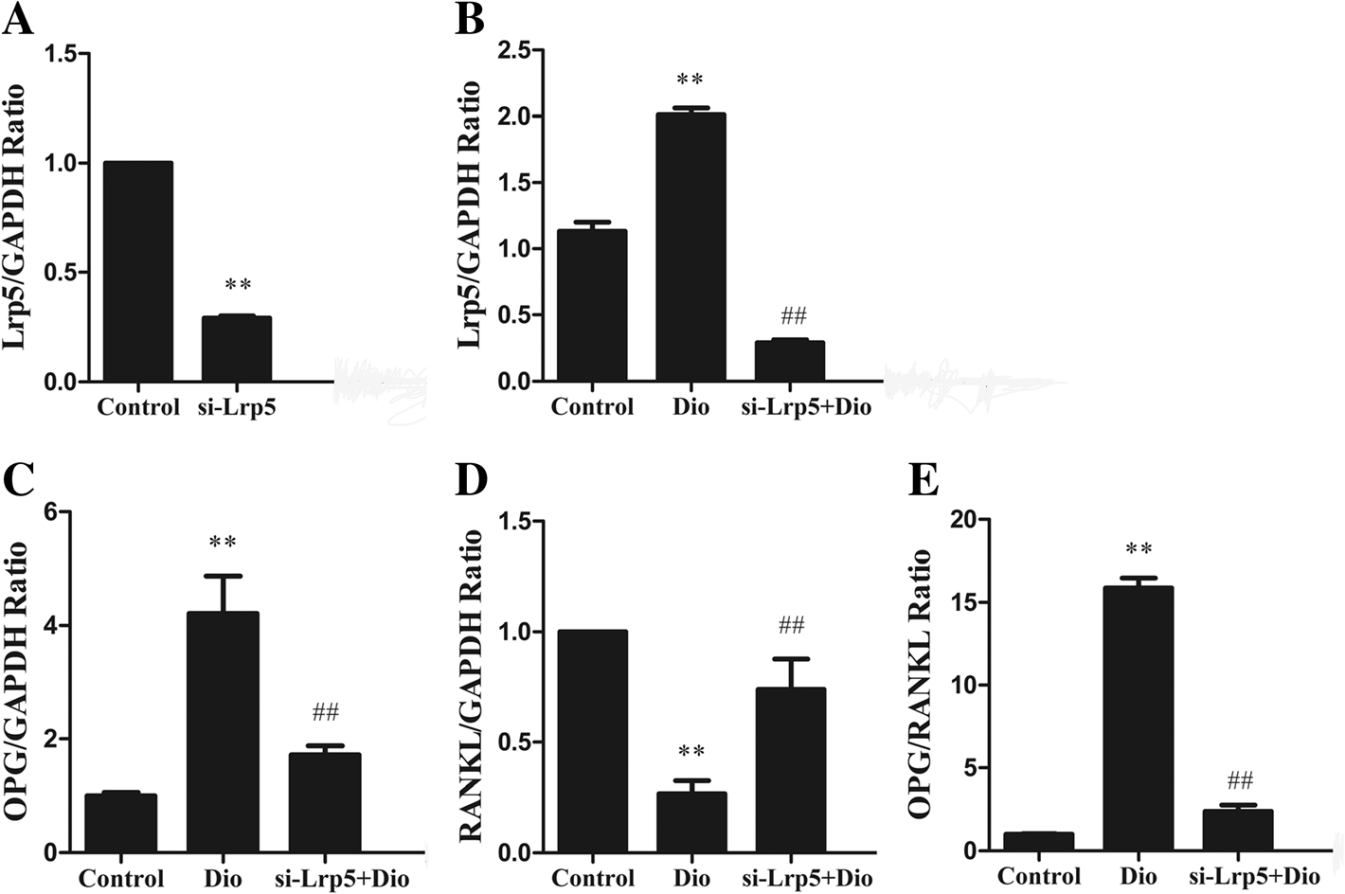
**The effect of dioscin increasing the OPG/RANKL mRNA ratio was dependent on Lrp5. (A)** Reduced Lrp5 expression by RNA interference against Lrp5. MC3T3-E1 cells grown in regular medium were transfected with Lrp5-siRNA construct and control vector. Total RNA was extracted and analyzed by RT-PCR. **(B)** The effect of dioscin-increased Lrp5 expression level in MC3T3-E1 cells which were transfected with Lrp5-siRNA was abolished. **(C, D, E)** Dioscin increased OPG/RANKL ratio was dependent on Lrp5 pathway. Cells were treated with vehicle and dioscin (1.0 μg/ml) for 72 h, and then the expression of Lrp5, OPG and RANKL mRNA were examined by RT-PCR. Results were obtained from three independent experiments and were expressed as mean ± SD. **P < 0.01 *vs* Control; ##P < 0.01 *vs* Dio group.

Then transfection with Lrp5-siRNA was used to prove that the effect of dioscin on the ratio of OPG/RANKL was dependent on Lrp5 pathway. MC3T3-E1 cells were transiently transfected with Lrp5-siRNA and control vector. The cells transfected with Lrp5-siRNA had an obvious reduction in the Lrp5 mRNA as demonstrated by RT-PCR (Figure 9A). To determine the effect of dioscin on the ratio of OPG/ RANKL in the cells with reduced Lrp5, we treated Lrp5-siRNA and control vector cells with 1.0 μg/ml of dioscin and determined the ratio of OPG/ RANKL by RT-PCR. As shown in Figure 9, dioscin treatment could not up-regulate the expression of Lrp5 mRNA (Figure 9B) and OPG mRNA (Figure 9C), decrease the expression of RANKL mRNA (Figure 9D) and increase OPG/ RANKL ratio (Figure 9E) in Lrp5-siRNA cells as in normal MC3T3-E1 cells (P < 0.01), indicating that the effect of dioscin on the OPG/RANKL mRNA ratio was partially dependent on Lrp5 pathway.

### The inducing effects of doscin on ALP activity, Lrp5, β-catenin, OPG/ RANKL gene expressions and β-catenin protein expression in MC3T3-E1 cells were dependent on the ER pathway

**Figure 10:**
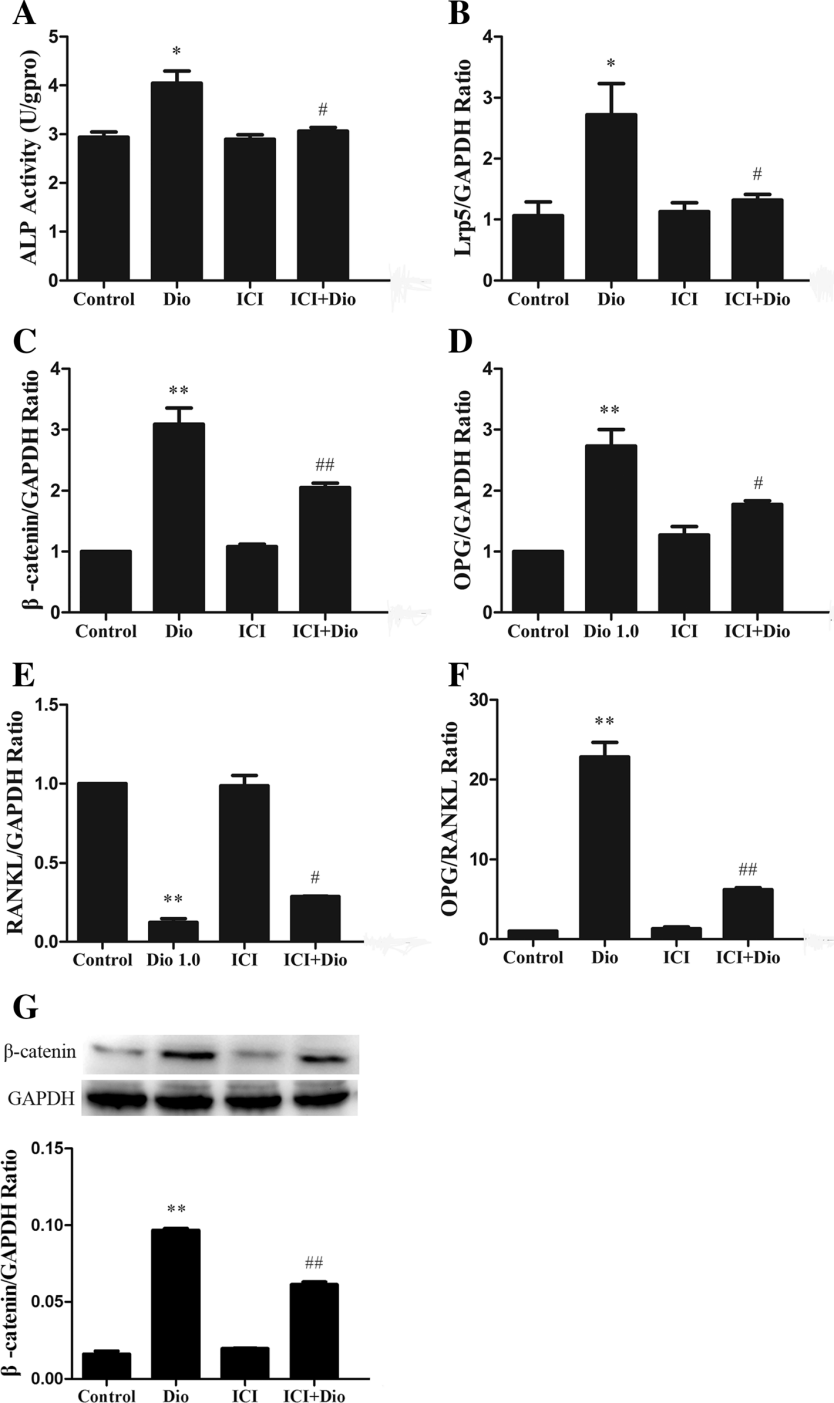
**Effects of ICI 182,780 on dioscin**-**induced ALP activity**, **Lrp5 and β**-**catenin mRNA expressions and ratio of OPG/RANKL mRNA in MC3T3**-**E1 cells.** MC3T3-E1 cells were treated with vehicle and dioscin (1.0 μg/ml) in the presence or absence of ICI 182, 780 (ICI). **(A)** ALP activity. Cell lysates were used for ALP activity measurement by an ALP activity assay kit. Results were obtained from three independent experiments and were expressed as mean ± SD. **(B)** Lrp5 mRNA expression level. **(C)** β-catenin mRNA expression level. **(D)** OPG mRNA expression level. **(E)** RANKL mRNA expression level. **(F)** OPG/RANKL mRNA ratio. **(G)** β-catenin protein expression level. Results were obtained from three independent experiments and expressed as mean ± SD. *P < 0.05; **P < 0.01 *vs* control. # P < 0.05; ## P < 0.01 *vs* Dio group.

In order to determine whether the stimulatory effects of dioscin on ALP activity, Lrp5, β-catenin, OPG/RANKL gene expressions and β-catenin protein expressions were dependent on the ER signaling pathway, MC3T3-E1 cells were co-incubated with ICI 182,780 (100 nM), an antagonist of both ER-α and ER-β. Then ALP activity was determined by ALP activity assay kit and Lrp5, β-catenin and OPG/RANKL gene expression were analyzed by RT-PCR. β-catenin protein expression was analyzed by Western blot. As shown in Figure 10A, 1.0 μg/ml of dioscin significantly increased MC3T3-E1 cell ALP activity (P < 0.05) and the stimulatory effect was abolished by co-treatment with ICI 182,780. Similarly, the stimulatory effects of 1.0 μg/ml dioscin on Lrp5 (Figure 10B), β-catenin (Figure 10C), OPG (Figure 10D) and RANKL (Figure 10E) as well as on the ratio of OPG/RANKL (Figure 10F) were also abolished by co-treatment with ICI 182,780. The effect of dioscin obviously increasing β-catenin protein expressions in MC3T3-E1 cell was also abolished by co-treatment with ICI 182,780 (Figure 10G). These results indicate that the stimulatory effects of dioscin on osteoblastic functions were ER-dependent.

## Discussion

This study evaluated the osteoprotective effects and mechanism of actions of dioscin in mouse pre-osteoblast like cells MC3T3-E1. We have demonstrated that dioscin is capable of promoting proliferation, differentiation and mineralization of osteoblasts and inhibiting osteoblasts apoptosis. ALP, representative of early stage of osteoblast differentiation markers, is known to be importantly involved in the initiation of mineralization during bone formation. And ALP activity is a critical indicator of osteoblasts differentiation and osteogenic properties [30,31]. Bcl-2 plays a crucial anti-apoptotic function role [27]. In our results, we revealed that dioscin could significantly increase ALP activity and up-regulate Bcl-2 expression level in MC3T3-E1 cells. Because MG-63 cell line has a similar antigenic prolife to that in primary cultured human osteoblasts from human bone tissue sections [32,33], therefore, we also detected the promoting effects of doscin on osteoblasts by using this human osteoblast-like cells. And the results indicated that dioscin could also promote the proliferation and differentiation of MG-63 cells significantly.

OPG and RANKL are osteoblast-derived proteins pivotal to the regulation of bone mass and play opposing effects on osteoclasts [34,35]. OPG, a decoy receptor for the RANKL, is expressed by osteoblasts. RANKL interacts with RANK on osteoclasts to stimulate bone resorption by increasing osteoclast differentiation, activation and survival. OPG can also bind to RANKL but prevents RANKL-RANK interaction [36], thus, inhibits bone resorption [35,36]. Therefore, the OPG/RANKL ratio is critical to the coupling of bone resorption to bone formation [35]. We found that OPG mRNA expression could be increased significantly (Figure 5A) and RANKL mRNA expression could be decreased significantly (Figure 5B) when MC3T3-E1 cells were exposed to various concentrations of dioscin. Therefore, we conclude that dioscin could promote osteoblasts proliferation by up-regulated the OPG expression and inhibit ostoclasts differentiation by decreased the RANKL expression.

ER signaling pathways play a crucial role in the bone remodeling, the development and maintenance of the skeleton [8,37,38]. Two ERs (ER-α and ER-β) have been reported to be differently expressed during osteoblast differentiation. And the view has also been accepted widely that estrogen acts on the bone cells through the classical ER-α and ER-β [10,38], and deficient of ER-α expression can result in osteoporosis [39]. And the human ER-β gene has also been reported to be associated with the risk of osteoporosis and bone mineral density (BMD) [40]. So ERs plays a significant role in the proliferation and differentiation of the osteoblasts, and ERs may be an important molecular target for treatment of osteoporosis and maintaining bone formation. In the present study, we have investigated that dioscin can up-regulate dose-dependently the expression of both ER-α and ER-β proteins (Figure 6, P < 0.01) in MC3T3-E1 cells. We also found that dioscin has the same effects in human osteoblast-like MG-63 cells. ICI 182,780 (Faslodex) from AstraZeneca (Cheshire, United Kingdom) is considered as a pure steroidal estrogen antagonist that was designed to be devoid of estrogen agonist action in both in vivo and in vitro models [41,42]. It can abolish estrogen agonist activity by competing with endogenous estrogen for ERs presented in the nuclei of estrogen responsive tissues [41,42]. As Figure 6B, E and Figure 6B, F shown, the expressions of ER-α (P < 0.05) and ER-β (P < 0.01) were blocked by ICI 182,780. At the same time, the effects of dioscin which stimulated ER-α and ER-β protein expression can be blunted by ICI 182, 780 (P < 0.01). And we found that the effects of doscin increasing ALP activity and the ratio of OPG/RANKL were also inhibited by ICI 182, 780. Therefore, we argue that dioscin may promote MC3T3-E1 cells proliferation and differentiation via the ER signaling pathway.

Wnt/β-catenin signaling pathway, is also critical in bone formation and maintenance of bone mass [43,44]. However, Lrp5, a critical co-receptor for Wnt signaling pathway and upstream of β-catenin, has been identified as an important contributor to bone health. And Lrp5 was observed to be associated with human HBM disease and OPPG syndrome characterized primarily by low bone mass through genetic studies of human bone abnormalities, Lrp5 knockin mice and Lrp5-deficient mice [26,29]. β-catenin signaling pathway plays an important role in bone formation in vivo [45,46] and deletion of the β-catenin gene can prevent osteoblast proliferation and differentiation in vitro [47]. Present study revealed that dioscin could increase obviously the expression level of Lrp5 mRNA, β-catenin mRNA and β-catenin protein in MC3T3-E1 cells. However, the effects of dioscin could be inhibited by ICI 182, 780. Therefore, our study suggests that the effect of dioscin regulating the expression level of Lrp5 and β-catenin might also be dependent on the ER signaling pathways.

Since Lrp5 also plays an important role in bone formation, then we will question the hypothesis: whether dioscin increases the ratio of OPG/ RANKL mRNA is dependent on Lrp5 signaling pathway? To demonstrate the hypothesis, the present study applies RNA interference to make Lrp5 gene in MC3T3-E1 cells be knocked down, then the cells were treated by dioscin (1.0 μg/ml) for 72 h. We found that the ratio of OPG/RANKL mRNA could not be up-regulated by doscin as in normal cells anymore. Thus, we conclude that dioscin performs its function, increasing significantly the ratio of OPG/RANKL mRNA, via Lrp5 signaling pathway partially.

In conclusion, the present study clearly demonstrated that dioscin not only could promote MC3T3-E1 cells proliferation and differentiation, but also could promote human osteoblast-like MG-63 cells proliferation and differentiation. And dioscin directly stimulated the expression level of Lrp5, β-catenin and increased the ratio of OPG/ RANKL. And these effects of dioscin might be dependent on ER pathway and Lrp5 pathway in MC3T3-E1 cells. Therefore, our results provide a new insight into the mechanisms of dioscin on prevention and treatment of osteoporosis.

## Conclusions

Our study provides the evidence to support the use of dioscin as an effective candidate for osteoporosis. Therefore, the results of our study indicated that dioscin has potential effects in prevention and treatment of osteoporosis.

## Abbreviations

Dio: Dioscin; ER-α: Estrogen receptor α; ER-β: Estrogen receptor β; Lrp5: Low-density lipoprotein receptor-related protein 5; ALP: Alkaline phosphatase; OPG: Osteoprotegerin; RANKL: Receptor activator of NF-κB ligand; RT-PCR: Reverse transcription- polymerase chain reaction; OPPG: Osteoporosis-pseudoglioma; HBM: High bone mass; HRT: Hormone replacement therapy.

## Competing interests

The authors declare that they have no competing interests.

## Authors’ contributions

HJS and MZL designed experiments, provided technological support and checked the manuscript. CFZ performed major experiments, data analysis and wrote the manuscript. SW and FX carried out the experiments and data analysis. JYP, YJ, CYW and KXL purified doscin and provided technological support. All authors have given final approval of the manuscript.
